# Clinical Reflections

**Published:** 1882-07

**Authors:** Ad. Lippe

**Affiliations:** Philadelphia


### CLINICAL REFLECTIONS.

AD. LIPPE, M. D., PHILADELPHIA.

Mr. H., age 68 years, a high liver, subject to attacks of gout, was suddenly attacked on the 30th of April, at 6 P. M., with what he thought was pains in the abdomen from indigestion; he took several doses of *Nux vomica*, but steadily becoming much worse requested me to call on him. I found him at 9 P. M., suffering intensely from hepatic colic. He was crying out with the pain, was very restless, could neither sit still or lie down, walked about from one end of the room to the other, then attempted to sit down, because he was exhausted, but could not stay in the same position for a minute; great thirst, but when he drank he was so nauseated that he had to vomit, and after throwing up the water, he vomited bile. His countenance showed great anxiety, and he expressed his conviction that this terrible pain would soon kill him. He received *one* dose of *Arsenicum alb.*$^{cm}$ (F.). The pain increased for about five minutes and then gradually became less; in half an hour he was able to lie down, and hot water cloths were then applied to the hepatic region; in another half an hour he fell asleep and passed a comfortable night. When I called next morning he had enjoyed a good breakfast, and complained only of great soreness in the hepatic region. On the third day he had a slight attack of pain, and another still slighter attack on the ninth day after his first very severe attack, the last ending in vomiting a great quantity of bile. The formerly clay-colored stools became normal and the almost black urine which he had passed in small quantities became also normal. He remains perfectly well, and has taken no medicine since I administered this one single dose of *Arsenicum*.

*Comments:* Homœopathy, sustained by modern progressive allopathic physicians, teaches us that we must treat *individuals*, not *diseases*. In the case above related, no doubt could exist as to the disease; it was a clear case of hepatic colic. The patient implored me to help him, he did not ask for Morphia or Laudanum but for "help." As a homœopathist, the only question that could possibly arise was, "*what is in this case and for the symptoms presenting themselves, the curative remedy*" and who, conversant with our Healing-Art and with the principles guiding us in our therapeutics, could, under these circumstances, have failed, at once, to recognize the great similarity of *Arsenicum* to the condition of the sufferer. The very expression of his countenance called for it, the intense restlessness driving him in utter despair and with great lamentations, from one position to another; the great thirst and the immediate vomiting after drinking, were such characteristic symptoms of the remedy that there could not be the slighest doubt as to its homœopathicity to the case. The first indications of its curative action was a clearing up of the countenance, bringing a more cheerful expression to his face. The result was "astonishing." The single dose of a highly potentized drug was all-sufficient to cure the sick. Astonishing, because the process by which such an infinitesimal dose acts on the sick organism is incomprehensible to our senses. These results still astonish us, even after witnessing them daily, as we do if we implicitly follow the strict rules laid down by the founder of our school, in his *Organon* of the Healing-Art; and what wonder if these frequently recurring confirmations of the great truths taught by Hahnemann make us scorn the substitutes offered by thoughtless men who desire to pervert, nay even attempt to misrepresent his teachings. If these men do not reach the same results, daily witnessed by the strict adherents to our school, it is their own fault. There are men who desire to lead us back again into, what they term, a more scientific application of the Law of the Similars, in which law they profess to believe, lead us back into fallacious hypotheses, lead us to first scientifically diagnosticate a case and then find a remedy which is known to cause symptoms similar to the pathological condition found on the sick. In the case above related they would be guided by the testimony of very learned men, who have in turn proclaimed *Arnica* and *China* or *Lycopodium* specifics for hepatic colic. No doubt one or the other of these remedies have cured that disease, *under certain circumstances*, but to draw the deduction from just one or even more cures that that remedy will cure all such cases is a fallacy, and the fallacy began when the healer attempted to prescribe for a form of a disease and forgot that he must prescribe for the individual and not for a disease; forgot that he pretends to practice Homœopathy and then blundered into inferior allopathy or eclecticism. Still there are others more censurable, homœopaths (as they have the effrontery to call themselves) who resort to eclectic treatment at once, who would not for the world be found guilty of administering an infinitesimal dose in so grave a case, who would not be guilty of trifling with people's lives in that way, but that they, more learned, will at once act like *humanitarians* and, therefore, at once pull out their hypodermic syringe and their precious solution of Morphia which they, though calling themselves homœopaths, *always* carry in their pockets. They administer it, and if the patient, after a few repetitions of such brainless applications, cease to have pain because he has ceased to live, *then* the humanitarian boldly declares that all that science could do for the sufferer had been done by men who are liberal, not bigoted. Fortunately the number of such men is a small one and they must or should know that they are spotted and despised both by the homœopathists and allopathists.

There is still another branch, to be sure a small one, but one which desperately demands full recognition as true homœopathists, who have fallen into the exploded errors of Mr. Lux, who, during

Hahnemann's day, tried to shed a new light on the Law of Cure, and desired to substitute the formula, *æqualia æqualibus curantur* for Hahnemann's formula, *similia similibus curantur*. Now what would one of these new lights have proposed to do in this grave case? Of course, true to the assertion by them made, "*that the product of a disease when very highly potentized will cure the disease itself*," a dose of highly potentized *Calculus hepaticus* would have been administered —no matter the varying chemical components of such calculi. This "fatal error" would be threefold: first, to prescribe for a disease, a pathological condition; second, to prescribe an unproved substance; third, to insist upon the necessity of administering a very highly potentized substance to secure a possible cure. As homœopathists, we treat individuals, not diseases, with remedies proved under the Law of the Similars. The posological question does not, *á priori*, come into consideration, though the most successful cures have been made with high potencies; and while it is admitted that potentization increases the curative powers of a drug, it is not to be forgotten that our knowledge of the sick-making powers of drugs was first obtained by provings made with crude or little potentized drugs, and that the first great victories of our Healing Art were gained by the administration of the lower and finally of the thirtieth potencies. To claim now that the change effected by attenuation is necessary for the higher Homœopathy, that dynamization by attenuation so alters every medicine and each individual centesimal attenuation as to make it "all the same but different;" that Syphilinum, for instance, becomes "the most like" to Syphilis in the CM attenuation, is to override all the fundamental principles of the homœopathic Healing Art and evidently must lead us into Lux's isopathy, which has proved itself to be "a fatal error." The case above related is only an additional evidence of the correctness of Hahnemann's teachings.

